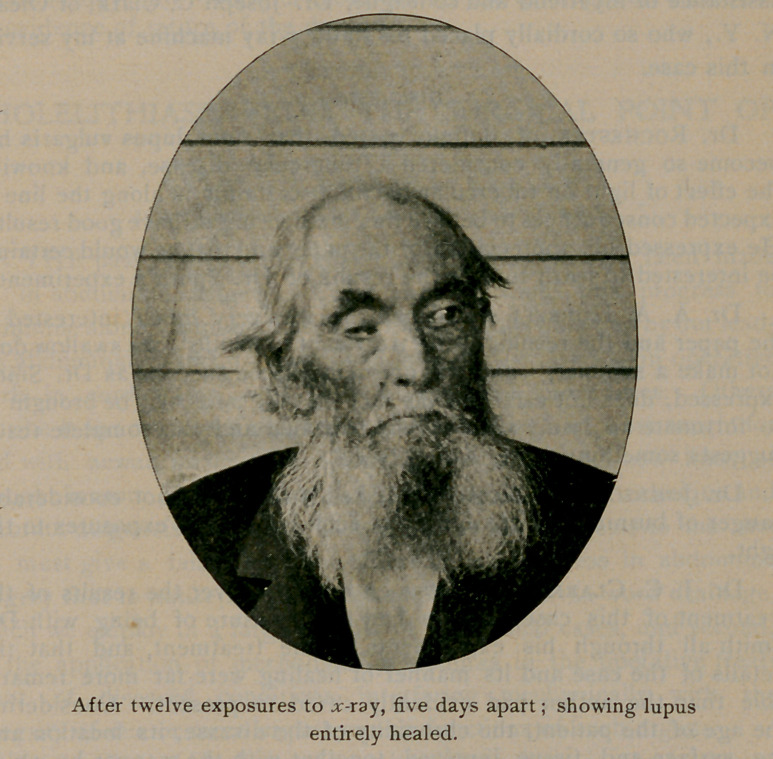# Lupus Vulgaris of Fifteen Years’ Standing Successfully Treated and Cured by Exposure to X-Ray1Read before the Cattaraugus County Medical Society, Salamanca, N.Y., November 20, 1900.

**Published:** 1901-01

**Authors:** A. Everett Smith

**Affiliations:** Olean, N. Y.


### BUFFALO MEDICAL JOURNAL.

VOL. XL.—LVI. JANUARY, 1901. No. 6.

### Original Communications.

### LUPUS VULGARIS OF FIFTEEN YEARS' STANDING SUCCESSFULLY TREATED AND CURED BY EXPOSURE TO *X*-RAY.[1]

By A. EVERETT SMITH, B. S., M. D., Olean, N. Y.

THE article which I am about to present today was prepared for the Olean Medical and Surgical Club, August 1, 1900. But since few heard the article at that time and as it has not yet appeared in print, your secretary has urged me to present it at this meeting.

That lupus vulgaris is a skin disease of the most persistent and incurable type, is evident to the student when he notices the long list of possible remedies for it, which our best authors recommend. That the pathology and treatment of the disease has been little understood, most practitioners of any great experience must admit when they recall their fruitless attempts to relieve these suffering patients. Pathological research points to tuberculosis as an etiological factor. Still, most of these cases have left one vivid impression on our minds by their failure to get well. Such, at least, has been my experience. After using the remedies and methods usually advised, I have seen the patient, uncured, drift on to others only to get similar results. To the conscientious physician such results are most humiliating and should spur him on to diligently seek other more successful treatment.

About two years ago, while making some experiments with bacteriological cultures, I observed the well-recognised fact, I believe, that cultures did not thrive so well when exposed to the light. About this time or later I noticed some experiments were being made by Dr. Finsen, of Copenhagen, Denmark, applying this principle in the treat-

1. Read before the Cattaraugus County Medical Society, Salamanca, N. Y., November 20, 1900.

ment of some bacteriological skin diseases. The light used, however, I believe, was high power arc electric light and the sunlight, both used with a powerful condenser. My understanding of the Finsen method is that the diseased surface is exposed to the direct rays of the light used at stated intervals, and that the results have been very favorable in many cases.

I was desirous of making some experiments with cases of lupus vulgaris, believing that by the use of the *x*-ray much better results could be obtained than by the arc electric rays or sunlight. I have accordingly the following case to report:

As nose appeared at beginning of treatment; showing lupous patch on nose of fifteen years' standing.

Frank Nichols, age about 80 years, of Shingle House, Penn., consulted me May 3, 1900, for an ulceration of the nose and face, which was involving his right eye. I found he had a lupus patch, which extended from the left side of the nose, going over the bridge and involving the right side of the nose, the inner canthus of the right eye and the inner thirds of the lids, together with the bulbous conjunctiva. He gave the following history: About fifteen years ago was slightly injured by a chip striking him on the bridge of the nose, breaking the skin. It never healed over completely but slowly increased in size, notwithstanding he had been treated by a score of physicians, a few skin specialists and numerous quacks. About a year ago it involved the eye, since which time it has progressed more rapidly in the mucous surfaces. There never was much pain from it, but it bled occasionally and disfigured him much. His general health and family history were good. No specific taint was elicited.

On commencing the treatment I took a photograph of his face, which I herewith submit, together with one taken June 10th, or about two months after the first. In both cases the negatives were not retouched, but show the skin just as it was.

After twelve exposures to *x*-ray, five days apart; showing lupus entirely healed.

I made a mask for the face, of sheet lead, cutting a hole for the nose and the diseased part of the right eye, and with this on exposed him about every fifth day for twenty minutes at a time, the diseased surface being placed about two inches from the light. He received in all twelve treatments. No medicine whatever was allowed. He was using applications of vaseline at first, but that was denied him. Marked improvement appeared after the second treatment, which was not interrupted until the sore was completely and entirely healed. After the second treatment healthy granulations appeared and healing was most remarkably speedy. There was no burning from the light or any other unpleasant symptoms complained of except a slight headache and a decided "crawling sensation" in the sore with the first two treatments. It will be noticed that the cicatrix has produced a slight ectropion by everting the inner end of the lower lid and has drawn the upper down and in, but vision is normal.

I do not claim priority in the use of *x*-ray in the treatment of lupus, though at this writing I have not noticed reports of any one else using it for this purpose. I wish to acknowledge the kindly assistance of my friend and colleague, Dr. Joseph C. Clark, of Olean, N. Y., who so cordially placed his static *x*-ray machine at my service in this case.

DISCUSSION.

Dr. ROCHESTER, of Buffalo, stated that since lupus vulgaris has become so generally considered a tubercular disease, and knowing the effect of light on tubercular bacilli, it certainly is along the line of expected consequences to believe this method might have good results. He expressed his appreciation of the paper and that he would certainly be interested to learn further the results of Dr. Smith's experiments.

Dr. A. A. HUBBELL stated that he was very much interested in the paper and the results of the treatment. While "one swallow does not make a summer," and this one remarkable success, as Dr. Smith expressed, does not establish the fact that all cases may be brought to so fortunate an issue, still the way it healed and the complete result suggests something more than accident.

Dr. JOHNSON, of Ellicottville, asked if there was not considerable danger of burning the epidermis by such prolonged exposures to the light.

Dr. J. C. CLARK said he was enthusiastic over the results of the treatment of this case, that he had the pleasure of being with Dr. Smith all through his experiments in the treatment, and that the details of the case and its manner of healing were far more remarkable than the author had evidently cared to present. Considering the age of the patient, the chronicity of the disease, its location and the surface and tissue involved, together with the manner by which it changed from a dirty sluggish ulcer to a healthy granulating surface, which healed so rapidly, were certainly most remarkable features. Certainly one case, no matter how successful, does not prove a proposition, but to have seen these experiments one cannot but conclude that they are very suggestive that the treatment will receive favor in the future.

Since Dr. Smith first reported this case I have been fortunate to be able to give the treatment a trial in another case: Mrs. H., aged about 60, of Olean, N. Y. She had a large angry lupus patch, involving the left ear and neck. Treatment was commenced September 28th last, using the same method as detailed by the writer. The results have been about the same, and at this time the lupus has almost entirely healed. This case was one of over five years' duration. Healthy granulations soon showed and healing has gone on very rapidly, until now only a small point remains, which I feel confident will be well in a few weeks.

Dr. SMITH stated he thought there was no danger of burning the skin if the static machine was used with a mild tube. He thought most severe burns reported came where the coil and a powerful tube were used. He further stated that he saw and examined his patient the other day, now over four months after the healing, and there was no evidence of return of the disease.

## Figures and Tables

**Figure f1:**
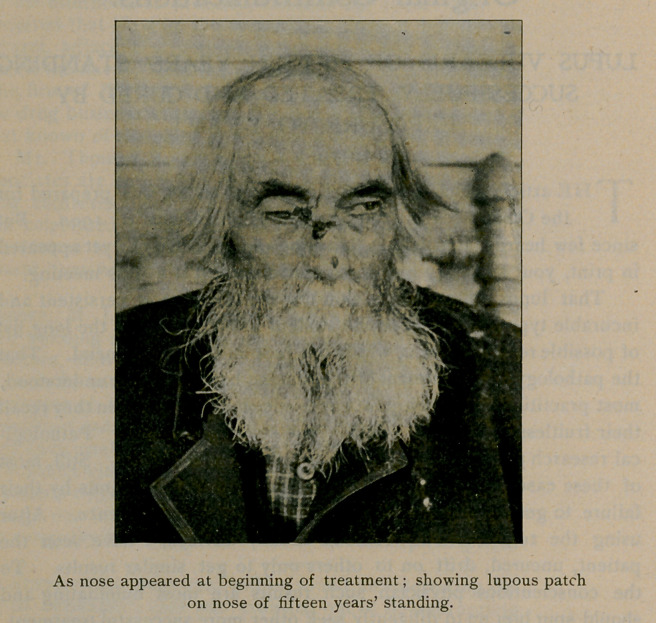


**Figure f2:**